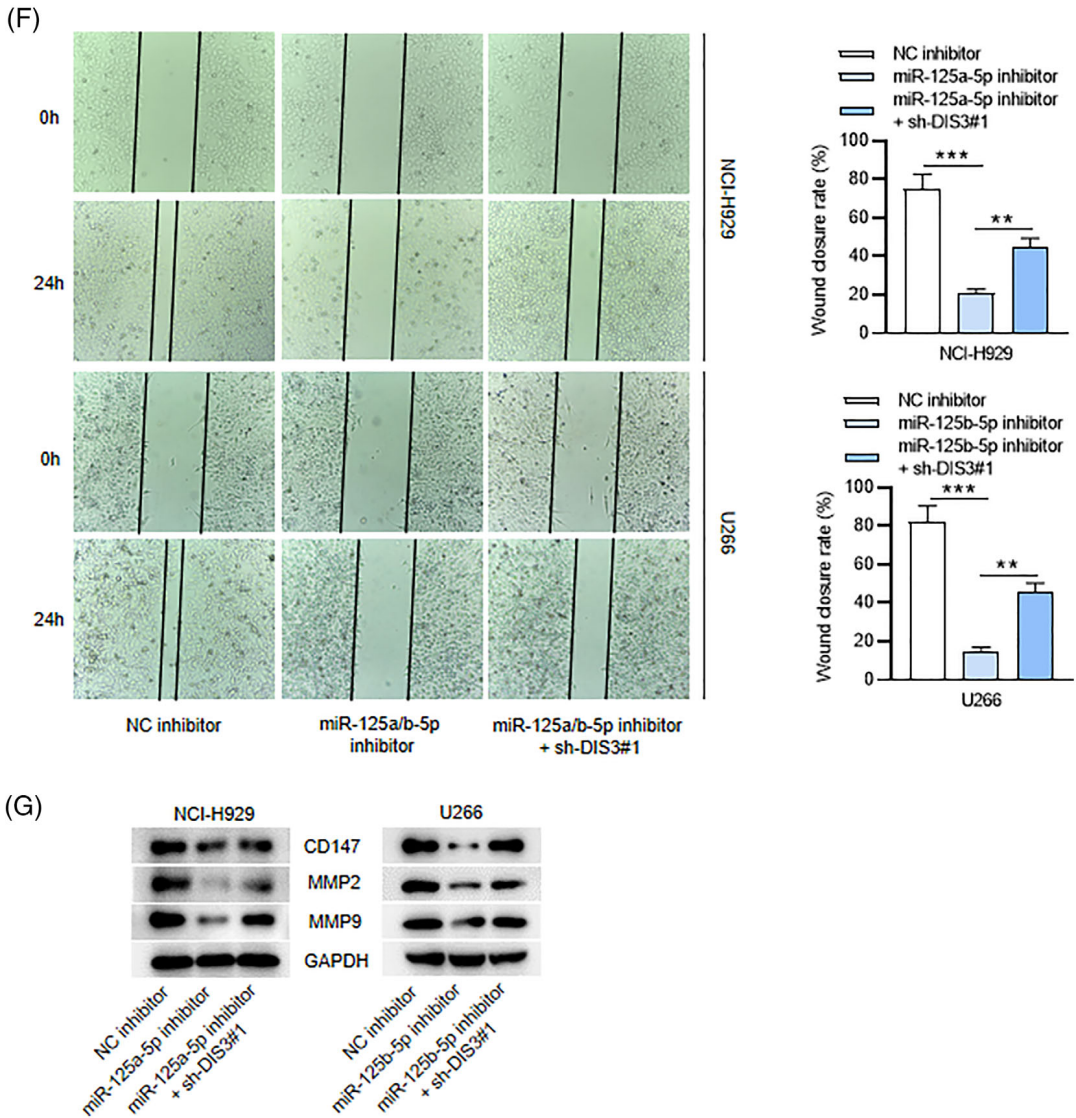# CORRIGENDUM

**DOI:** 10.1002/kjm2.12609

**Published:** 2022-10-10

Corrigendum to the article “MicroRNA‐125a/b‐5p promotes malignant behavior in multiple myeloma cells and xenograft tumor growth by targeting DIS3. Zhang T, Wang L‐L, Guan J, Zhou Y, Cheng P, Zou L. Kaohsiung J Med Sci. 2022;38(6): 574–84. https://doi.org/10.1002/kjm2.12534, the author has found errors in the Figures 1 and 3.

The data for Transwell assays were incorrectly arranged. The changes did not affect the final conclusions. The updated Figures l and 3 is shown below.

Updated Figure 1:

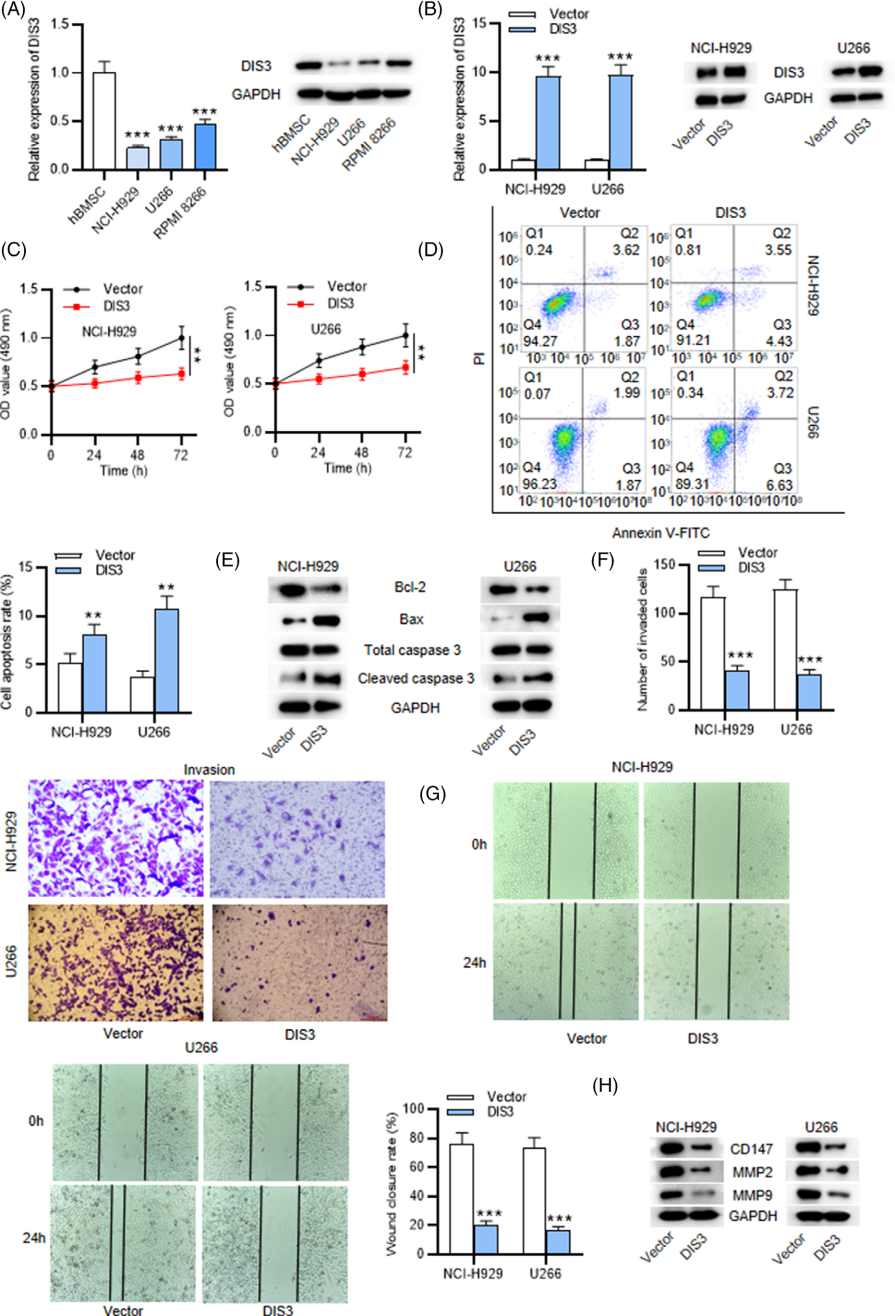

Updated Figure 3–1.

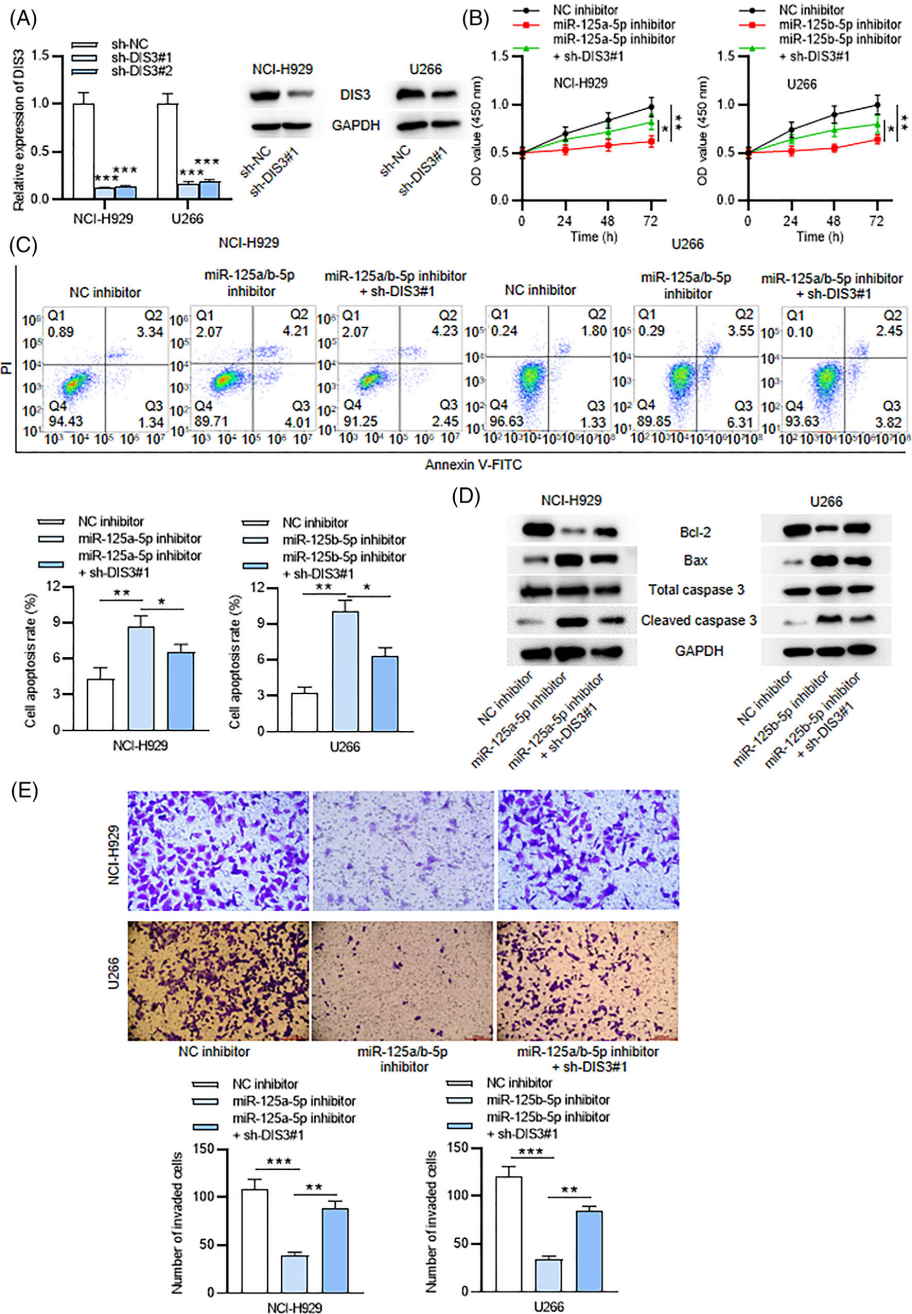

Updated Figure 3–2.